# Arbuscular Mycorrhizal Fungi Mediated Alleviation of Drought Stress via Non-Enzymatic Antioxidants: A Meta-Analysis

**DOI:** 10.3390/plants11192448

**Published:** 2022-09-20

**Authors:** Murugesan Chandrasekaran

**Affiliations:** Department of Food Science and Biotechnology, Sejong University, 209 Neundong-go, Gwangjin-gu, Seoul 05006, Korea; chandrubdubio@sejong.ac.kr; Tel.: +82-2-3408-4026

**Keywords:** abscisic acid, arbuscular mycorrhizal fungi, drought stress, hydrogen peroxide, malondialdehyde, meta-analysis

## Abstract

Drought stress constrains plant cell metabolism and induces the production of reactive oxygen species (ROS). In response to drought stress, plants induce a series of physiological and biochemical changes, scavenging ROS. Among soil microbes, arbuscular mycorrhizal fungi (AMF) are found to be effective ameliorators of ROS under drought-stress conditions. However, the comprehensive roles of the oxidative stress ameliorators mediated by AMF in alleviating drought stress are not studied in detail. The present study aims to determine the oxidative stress ameliorators using meta-analysis highlighting AMF inoculation efficacy on drought stress alleviation. The results confirmed that AMF inoculation had a significant reduction in hydrogen peroxide (H_2_O_2_), malondialdehyde (MDA), and electrolyte leakage (EL). Nevertheless, proline accumulation was found to have a non-significant correlation with AMF inoculation. Further, carotenoids and soluble sugars increased positively in AMF-inoculated plants under drought stress and there was a subsequent reduction of abscisic acid (ABA). The results of the meta-analysis reveal the benefits of AMF inoculation with reduced H_2_O_2_ levels leading to reduced lipid peroxidation (MDA) and increased membrane stability (EL). Thus, the present assessment reveals the sequence of events involved in eliciting drought stress alleviation due to AMF inoculation.

## 1. Introduction

Drought represents the alarming global abiotic stress phenomenon disturbing production and productivity in agriculture [1,2,3]. The ever-increasing population addresses the efficient use of dehydrated soils in drought-prone regions. Drought stress resilience mechanisms are unavoidable even in non-arid regions owing to seasonal changes [3,4,5]. Drought stress like other abiotic stress factors causes metabolic imbalance due to the accumulation of reactive oxygen species (ROS) including hydroxyl radicals (OH●), hydrogen peroxide (H_2_O_2_), singlet oxygen (^1^O_2_), and superoxide radicals (−O^2^) [3,4,6,7]. However, upon culmination of ROS in cells leading to phytotoxic levels, ROS initiates oxidative cascades in an uncontrolled manner. However, when reaching a certain level of phytotoxicity, ROS becomes extremely deleterious, initiating uncontrolled oxidative cascades that damage cell membranes and other cellular components resulting in oxidative stress and eventually programmed cell death [3,5,8]. Subsequently, ROS accumulation triggers stress defense responses [8]. The antioxidant system is considered to be a good candidate for the elimination of the excess accumulation of ROS and protect plant metabolism [4,6]. As a whole, the antioxidant mechanisms and osmotic regulators are attributed to quenching the enormous accumulation of ROS, thereby stimulating plant metabolism and homeostasis [4,6]. 

Hence, various enzymatic and non-enzymatic defense reactions are emancipated in obtaining cellular homeostasis as an oxidative stress abatement mechanism that accounts for oxidative stress reduction [1,3,5]. One of the most damaging oxidative effects is the peroxidation of membrane lipids, which results in the concomitant production of malondialdehyde (MDA) [9]. Moreover, plants accumulate osmolytes such as proline (PL), and soluble sugar (SS) during drought stress, which protect plants from dehydration and oxidative injury [10]. Proline, an amino acid, plays a very valuable role in plants exposed to various stress conditions including drought stress. Also acting as an essential osmolyte, proline plays three key roles during stress, that is, an antioxidative defense molecule, a metal chelator, and a signaling molecule. Among these regulated physiological responses, the plant hormone abscisic acid (ABA) plays a central role. Although rapid production of ABA in response to drought is essential to define ABA as a stress hormone, equally rapid catabolism of ABA is also essential when such stresses are reduced for redox homeostasis [11,12]. Hence, the accumulation of ABA and H_2_O_2_ in plants plays a crucial role in ROS scavenging activity during water stress [7,11]. 

Several reports have shown that microbial activity in the rhizosphere plays an important role in drought-induced antioxidant responses since it can alleviate the effects of drought stress in plants [13,14,15,16,17]. Among soil microbes, arbuscular mycorrhizal fungi (AMF), which associate with the roots of most terrestrial plants, may stimulate the growth of plants and contribute to enhancing plant tolerance to drought stress by enhancing plant processes, such as nutrient acquisition, photosynthesis, root formation and stress resistance [15,17,18]. AMF harbors several enzymatic antioxidants and non-enzymatic antioxidants mitigating oxidative bursts in response to ROS accumulation [19,20,21]. Experimental intensity and duration of drought stress have modified the host plants’ response and plant performance [22,23,24]. Similarly, variations have been observed amongst various plant species [13,14,16,25]. In addition, AMF inoculation was shown as an alternative to escape drought stress conditions. In this sense, the higher membrane stability is often related to lower MDA levels because of lipid peroxidation. Previous studies also showed a reduction in H_2_O_2_ and MDA in AMF-inoculated plants [17,18,19,20,21,22,23,24,25]. Proline content has also been reported to vary among mycorrhizal plants, and thus, it may serve as a parameter to evaluate the effects of AMF and drought stress on plants. Hence, the present meta-analysis was preceded in summarizing the plant responses during drought stress with AMF inoculation. The representative indices were grouped into plasma membrane permeability (ROS, EL, MDA), and non-enzymatic antioxidants (ABA, proline, carotenoids, and soluble sugars) based on the morphology, physiology, and functionalities of plants. The meta-analysis was performed employing necessary data from previous reports involving AMF inoculation effects in answering the below-listed key questions in deciphering drought stress sequel: 1. Do AM fungi inoculation contributes to drought stress management by changes in non-enzymatic antioxidant production? 2. Does the magnitude of production vary among different non-enzymatic anti-oxidants under drought stress? 3. If the magnitude is established, is it stress-dependent? 4. Does species or genus richness of AM fungi affect the magnitude of drought stress alleviation?

## 2. Materials and Methods

### 2.1. Data Collection

Peer-reviewed publications were collected from the Web of Science and Google Scholar, till 2021, and a database was created for further meta-analysis. The following search terms were combined: AMF or AM fungi* or mycorrhiza* or mycorrhizal inoculation* or AMF inoculation* and drought stress or water stress* and non-enzymatic antioxidant*, MDA, Proline* and stress alleviation or stress mitigation*. The use of the Boolean truncation (‘*’) character ensured that the variations of the word such as mycorrhizae, mycorrhizas, mycorrhizal, and so on were included. All publications were screened for suitability for our analysis. SEs from published papers were converted to SD by SE * sqrt (N) using a meta-win 2.0 statistical calculator. WebPlotDigitizer was used to tabulate data after direct extraction and graphical representation of the extracted data. Moreover, AMF species, plant species, plant functional group (herbaceous, woody, and grass), taxonomical group (monocot and dicot), and experimental conditions were collected directly from the publications. Data was collected from plants based on response variables, instead of separate organs/parts to avoid bias in our analysis. Among AMF species studied for the analysis of single AMF inoculum effects, we used the two most studied AMF species, *Rhizophagus irregularis* and *Funneliformis mosseae*, for comparative analysis of response variables. We used field capacity data of published articles as such for differentiation of the level of drought stress. We divided drought stress into three levels based on the field capacity, mild (60–80% field capacity), moderate (40–60% field capacity), and severe (0–40% field capacity) drought stress based on the information in the published research papers.

### 2.2. Inclusion Criteria

**Figure 1:**
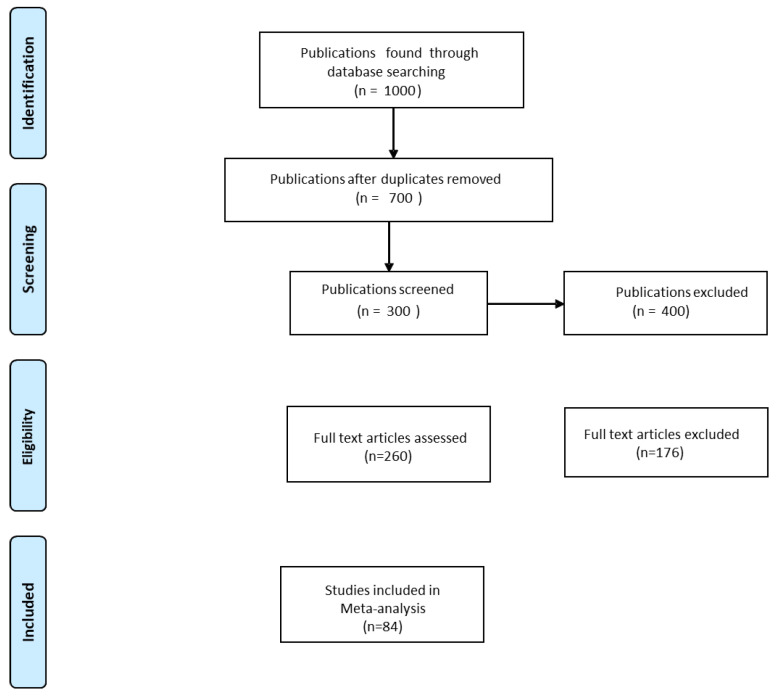
Flow diagram of meta-analysis.

These searches resulted in 700 published and unpublished online references, of which 260 were considered likely to contain significant information. Data were collected from 260 publications. To avoid bias in the selection of published papers, selected data were based on the following criteria: (1) Published (International publications) papers should contain experiments with both control (Without AMF inoculation) and treatment (with AMF inoculation) data; (2) publications should have means, standard deviations (SD), or standard errors (SE) with replication numbers; (3) publications should have the level of drought intensity (percentage of field capacity); (4) a minimum of two extractable observations from each publication; (5) excluded studies with single observations; (6) publications were excluded when the study was based on tolerant or sensitive genotypes; (7) publications were excluded based on polyethylene glycol (PEG) induced drought stress studies; (8) publications were excluded studies containing data other than antioxidant enzymes. Based on our inclusion criteria 176 publications were excluded, and the list was refined to 84 publications (Figure 1, Table S1-Dataset, Table S2). 

From the 84 publications, 518 observations were identified for the meta-analysis of oxidative stress indicator’s response to AMF inoculation under drought stress. The pooled data from 84 research publications comprise 50 plant species excluding mutant species, tolerant or sensitive species, and 18 AMF species. The representative oxidative stress indicators were fixed into 124 observations for MDA, 127 observations for proline, 40 observations for ABA, 33 observations for EL, 39 observations for carotenoids, 79 observations for soluble sugars, and 75 observations for H_2_O_2_. Three drought intensities, namely mild stress based on 84 observations from 23 published papers, moderate stress based on 232 observations from 61 published papers, and severe stress based on 201 observations from 43 published papers, were evaluated. Thus, the different oxidative stress indicator responses under different stress levels in AMF-inoculated plants were evaluated through meta-analysis.

### 2.3. Data-Analysis

Meta-win v2.0 software (Version 2.0; Rosenberg, Sunderland, MA, USA) was utilized for the estimation of weighted mean effect sizes across various studies and 95% confidence intervals (CIs) of values depending on various effect sizes and variance incidence of pertinent individual research were considered. A modified method by Hedges et al. [26] and Rosenberg et al. [27] was used in antioxidant efficiency evaluation and the responses of antioxidant enzymes and H_2_O_2_ dynamics due to the priming of AMF symbiosis abating drought stress. lnR-size/response ratios (the natural log of the ratio of the mean value of a variable of interest) representing the variable effects of drought stress due to AMF inoculation than non-inoculated plants were used. The magnitude of inoculation effects is depicted using the formula: lnR = ln (Xi/Xc) = lnXi – lnXc. Where Xi and Xc correspond to the response variable values differentiating the individual observation between the treatment and control, appropriately. Sampling variance for each lnR was calculated using the corresponding values based on sample sizes, following the equation: vlnR = (1/Ni) * (Si/Xi) + (1/Nc) * (Sc/Xc). Where Ni, Nc, Si, and Sc denote the sample sizes, standard deviations in the experimental, and control groups, respectively. Homogeneity variance statistic Q factor corresponding to non-categorical analyses was estimated employing chi-square distribution (*p* < 0.05). For each categorical analysis, the total heterogeneity was calculated among studies (Q_T_) within group heterogeneity (Q_W_), or between group heterogeneity (Q_B_). Studies were considered significant when QB was significant (*p* < 0.05) and described at least 10% of the total variation (Q_B_/Q_T_ ≥ 0.1) [10]. Zero (0) effect sizes suggest no difference in effects between the experimental and control groups, negative values represent reduced AMF inoculation effects where the control group achieves a healthier response than the experimental group, and positive values represent increased effects in response to AM fungi inoculation where the experimental group reaches a healthier response than the control group. The percentage efficiency of AMF inoculation was calculated employing (exp (lnR) − 1) * 100%. The percentage was compared to control and statistical significance at *p* < 0.05 values are rendered statistically significant. 

## 3. Results

### 3.1. Overall Analysis

**Figure 2:**
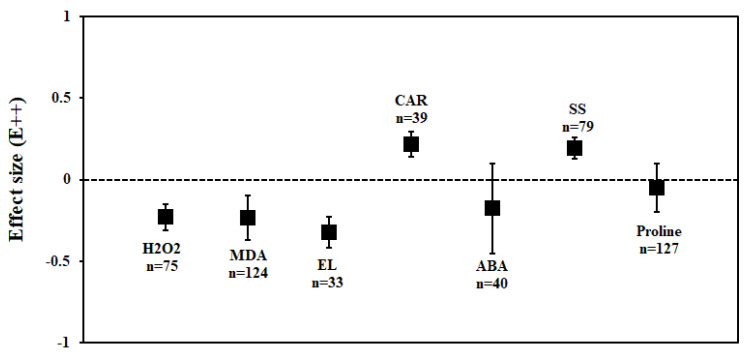
Effect of arbuscular mycorrhizal inoculation responses on non-enzymatic antioxidants under drought stress. Error bars are means ± 95% confidence intervals (CIs). Where the CIs do not overlap the horizontal dashed lines, the effect size for a response variable is significant at *p* < 0.05. The numbers of observations are shown above the bar. Note: H_2_O_2_–Hydrogen peroxide, MDA–Malondialdehyde, EL–Electrolyte leakage, CAR–-Carotenoids, ABA–Abscisic acid, SS–Soluble sugars.

Overall, the inoculation with AMF had significant positive effects on plant performance and these effects were maintained under drought stress conditions. In particular, the inoculation with AM fungi significantly reduced H_2_O_2_ production by 21% under drought stress conditions when compared to non-mycorrhizal (NM) plants. In addition, as compared to NM plants the inoculation with AM fungi significantly reduced MDA and EL by 21% and 29%, respectively, under drought stress, which in turn-maintained membrane stability. Oxidative stress markers such as ABA and proline content were reduced by 11% and 2%, respectively, but had no significant impacts on AMF inoculation. Furthermore, inoculation with AM fungi increased carotenoids and soluble sugar content by 32% and 28%, respectively (Figure 2, Table 1).

Different levels of drought stress significantly (*p* < 0.001) changed the AM effect on the oxidative stress ameliorator levels in crop plants. Among levels of stress, mild stress showed an increased (12%) AM effect, whereas both moderate and severe stress showed decreased (6% and 19%, respectively) AM effect. Different AM taxa showed a broad range of AM effects in crop plants under drought stress conditions. The highest drought stress reduction was achieved by *Diversispora versiformis* and *Claroideoglomus etunicatum*. However, the drought stress reduction by *R. irregularis* and *F. mosseae* was less pronounced but still significant. Moreover, mixed species showed more negative responses than those of single species inoculation under drought stress. Further, categorical analyses were conducted to explore AM responses for individual crop plants. Our results showed significant positive effects on plant family and plant species (*p* < 0.001) indicating the effectuating benefits of AMF inoculation under drought stress conditions.

### 3.2. H_2_O_2_ Production

**Figure 3:**
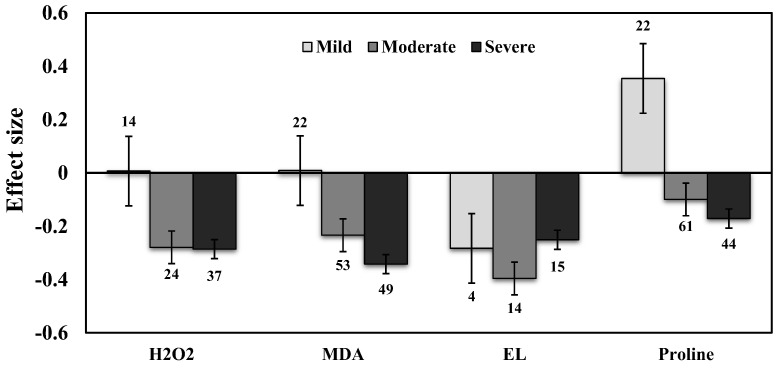
Effect of drought stress on H_2_O_2_, MDA, EL, and Proline levels in AMF inoculated plants. Error bars are means ± 95% confidence intervals (CIs). Where the CIs do not overlap the horizontal lines, the effect size for a response variable is significant at *p* < 0.05. The numbers of observations are shown above the bar. Note: H_2_O_2_–Hydrogen peroxide, MDA–Malondialdehyde, EL–Electrolyte leakage.

Separate meta-analyses were conducted for the H_2_O_2_ for sorting the AMF inoculation and ROS accumulation under drought stress conditions. AMF inoculation significantly reduced the H_2_O_2_ concentration in all plants under drought stress (−21%, *p* < 0.01) compared to those of NM plants. Further, analyzed the importance of the level of drought intensity on H_2_O_2_ production in AMF inoculated plants. Results showed that the effect size of H_2_O_2_ increased by 1% for mild drought stress intensity when compared to non-inoculated controls but was found to be non-significant. Whereas, for moderate and severe stress the effect sizes decreased significantly by −24% and −25%, respectively (*p* < 0.01) (Figure 3). 

**Figure 4:**
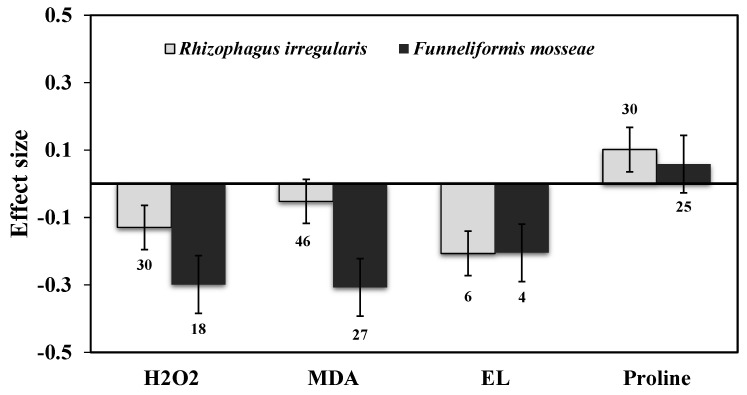
Categorical analysis of AMF species on H_2_O_2_, MDA, EL, and Proline levels. Error bars are means ± 95% confidence intervals (CIs). Where the CIs do not overlap the horizontal lines, the effect size for a response variable is significant at *p* < 0.05. The numbers of observations are shown above the bar. Note: H_2_O_2_–Hydrogen peroxide, MDA–Malondialdehyde, EL–Electrolyte leakage.

**Figure 5:**
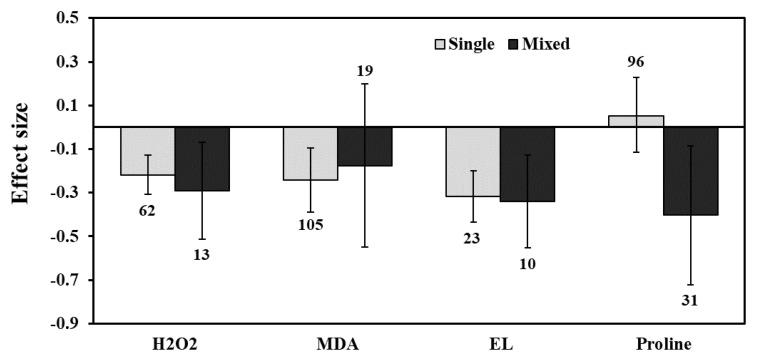
Categorical analysis of AMF inoculation type on H_2_O_2_, MDA, EL, and Proline levels. Error bars are means ± 95% confidence intervals (CIs). Where the CIs do not overlap the horizontal lines, the effect size for a response variable is significant at *p* < 0.05. The numbers of observations are shown above the bar. Note: H_2_O_2_–Hydrogen peroxide, MDA–Malondialdehyde, EL–Electrolyte leakage.

**Figure 6:**
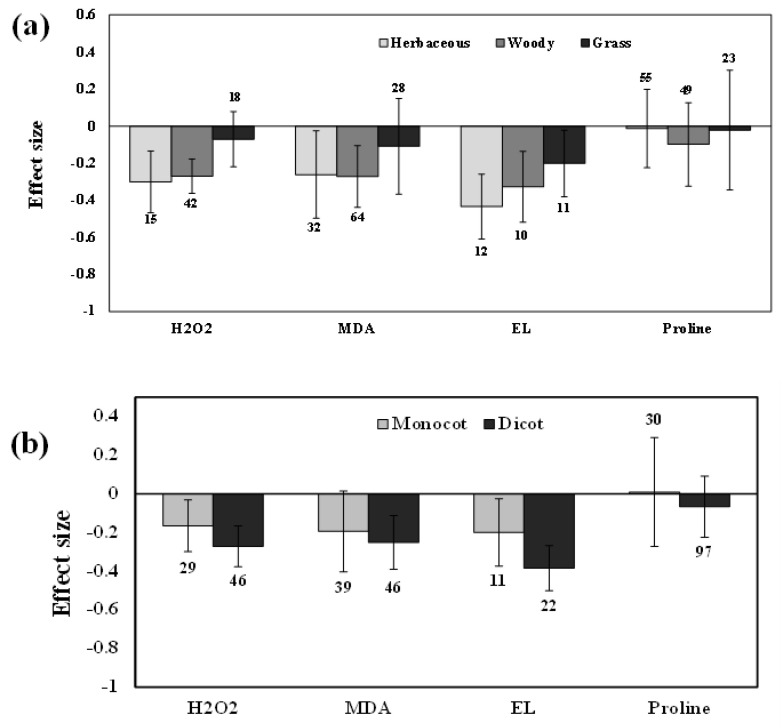
Categorical analysis of plant functional groups (**a**) and taxonomic groups (**b**) on H_2_O_2_, MDA, EL, and Proline levels. Error bars are means ± 95% confidence intervals (CIs). Where the CIs do not overlap the horizontal lines, the effect size for a response variable is significant at *p* < 0.05. The numbers of observations are shown above the bar. Note: H_2_O_2_–Hydrogen peroxide, MDA–Malondialdehyde, EL–Electrolyte leakage.

Categorical analysis showed significant positive effects on AMF species and AMF inoculation. Among AMF species, *F. mosseae* showed a more negative response for H_2_O_2_ than those of *R. irregularis* under drought stress (Figure 4). Among AMF inoculation, mixed inoculation was found to be a little more negative than single-species inoculation (Figure 5). When compared within each plant’s functional groups, H_2_O_2_ accumulation was more reduced for herbaceous plants than woody and grass plants under drought stress in AMF inoculated plants (Figure 6a). In addition, among the taxonomical group, dicot plants showed the highest effect size than monocot plants (Figure 6b).

### 3.3. Lipid Peroxidation and Electrolyte Leakage

The effect size of MDA increased by 1% for mild stress but decreased under moderate and severe drought stress, by 21% and 39%, respectively (*p* < 0.01) (Figure 3). Categorical analysis of AMF species showed a significant response for *F. mosseae* but *R. irregularis* showed a non-significant response whose confidence interval overlapped with zero (Figure 4). Moreover, single-species inoculation had a significant response for MDA but mixed-species inoculation showed a non-significant response whose confidence interval overlapped with zero (Figure 5). Among plant functional groups, we found a similar reduction in both herbaceous and woody plants, whereas grass was found to be non-significant whose confidence interval overlapped with zero (Figure 6a).

Our results showed that the effect sizes of EL significantly decreased under drought stress (n = 32, E++ = −0.32, CI = −0.42 to –0.23, *p* = 0.01). When considering drought intensity, we found that effect sizes of moderate stress showed a more negative response (33%), compared to those of severe stress (22%) (Figure 3). When dividing the plant species into three growth forms (herbaceous, woody, and grass), we found that herbaceous plants showed a more negative response to EL than woody and grass plants (Figure 6a). When the two plant taxonomical groups (monocot and dicot) were considered, we found the effect sizes of monocot plants had less negative for EL than those of dicot plants under drought stress in AMF inoculated plants (Figure 6b). Among AMF species, *F. mosseae* and *R. irregularis* showed a similar response to EL under drought stress (Figure 4). AMF richness also showed a similar response for both single- and mixed-species inoculation under drought stress (Figure 5).

### 3.4. ABA Content

**Figure 7:**
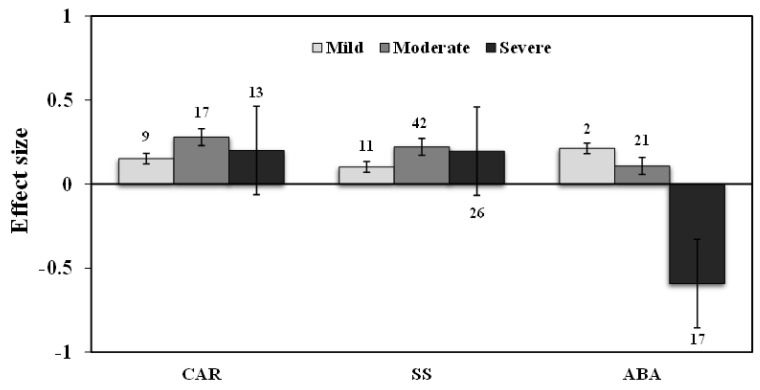
Effect of drought stress on CAR, SS, and ABA levels in AMF inoculated plants. Error bars are means ± 95% confidence intervals (CIs). Where the CIs do not overlap the horizontal lines, the effect size for a response variable is significant at *p* < 0.05. The numbers of observations are shown above the bar. Note: CAR–Carotenoids, SS–Soluble sugars, ABA–Abscisic acid.

Across all observations, AMF inoculation showed decreased ABA level by 16% under drought stress, but non-significant (n = 40; E++ = −0.18; 95 % CI, −0.45 to 0.09), where 95% CIs slightly overlapped with zero (Figure 2). Drought intensity levels showed that the effect sizes of ABA increased by 24% and 11%, respectively, for mild stress and moderate drought stress intensity. Whereas, the effect sizes of severe stress decreased by 45% under drought stress (Figure 7). 

**Figure 8:**
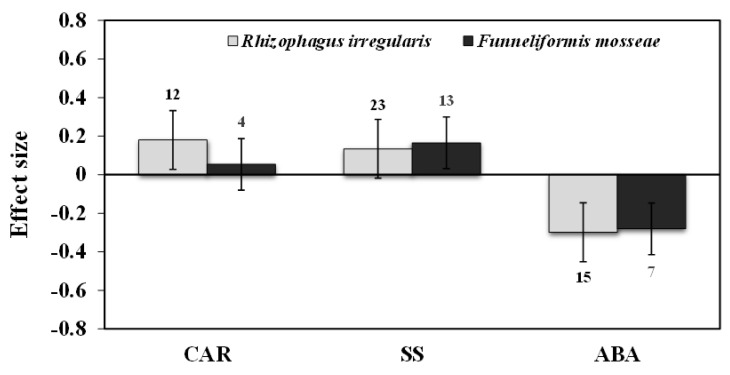
Categorical analysis of AMF species on CAR, SS, and ABA levels. Error bars are means ±95% confidence intervals (CIs). Where the CIs do not overlap the horizontal lines, the effect size for a response variable is significant at *p* < 0.05. The numbers of observations are shown above the bar. Note: CAR–Carotenoids, SS–Soluble sugars, ABA–Abscisic acid.

**Figure 9:**
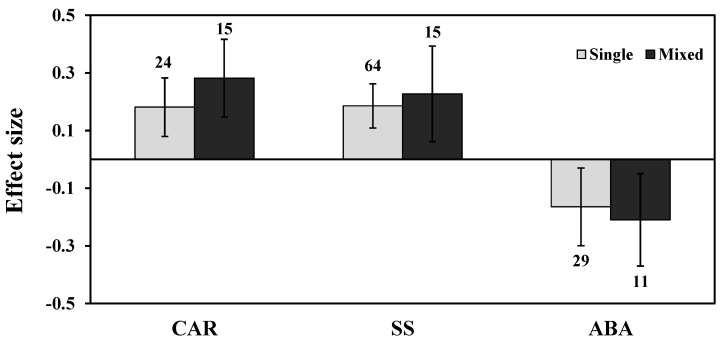
Categorical analysis of AMF inoculation type on CAR, SS, and ABA levels. Error bars are means ± 95% confidence intervals (CIs). Where the CIs do not overlap the horizontal lines, the effect size for a response variable is significant at *p* < 0.05. The numbers of observations are shown above the bar. Note: CAR–Carotenoids, SS–Soluble sugars, ABA–Abscisic acid.

**Figure 10:**
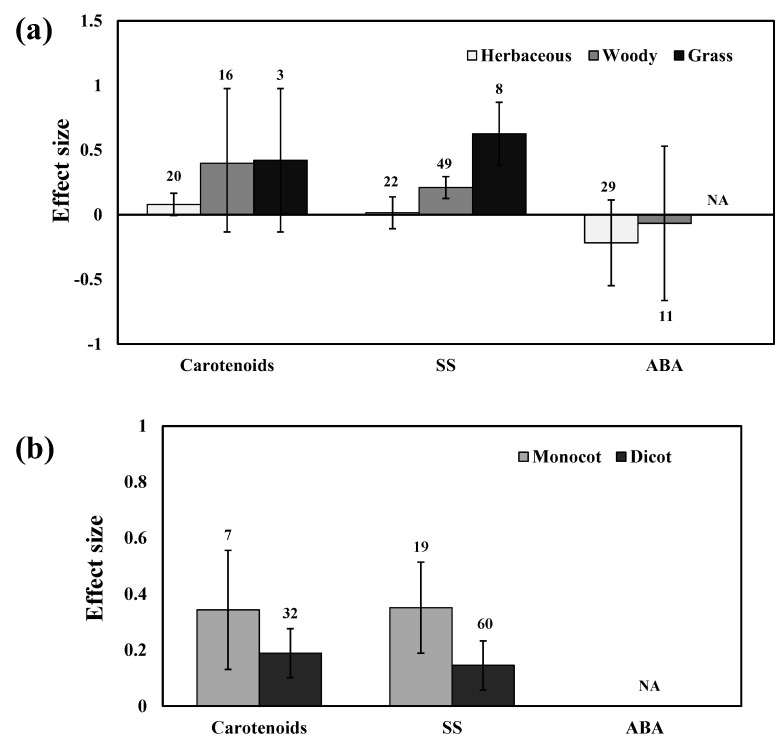
Categorical analysis of plant functional groups (**a**) and taxonomical groups (**b**) on CAR, SS, and ABA levels. Error bars are means ± 95% confidence intervals (CIs). Where the CIs do not overlap the horizontal lines, the effect size for a response variable is significant at *p* < 0.05. The numbers of observations are shown above the bar. Note: CAR–Carotenoids, SS–Soluble sugars, ABA–Abscisic acid.

Among most studies of AMF species, both *R. irregularis* and *F. mosseae* showed negative responses under drought stress for ABA levels (Figure 8). Categorical analysis of AMF inoculation indicated that single-species inoculation showed less negative (15%) than those of mixed species (19%) for ABA (Figure 9). Among plant functional groups, herbaceous plants were found to be more negative than woody plants but were found to be non-significant as their confidence interval overlapped with zero (Figure 10).

### 3.5. Carotenoids and Soluble Sugars

AMF inoculation significantly increased carotenoid levels by 28% compared to those of non-inoculated plants under drought stress. Moreover, significant variations among studies was observed in almost all studies (n = 39; E++ = 0.22; CI = 0.14 to 0.29; *p* < 0.0001). Among different stress levels, moderate stress increased the carotenoids and soluble sugar content by 32%, and 25%, respectively (Figure 7). Whereas mild stress increased by 16%, and 10%, respectively, for carotenoids and soluble sugars. Severe stress increased both carotenoids and soluble sugars by 22%. Among AMF species, *R. irregularis* showed a more positive response than *F. mosseae* for carotenoids. Whereas, *F. mosseae* showed a more positive response of *R. irregularis* for soluble sugars (Figure 8). Moreover, mixed inoculation had more positive for both carotenoids and soluble sugars than single-species inoculation (Figure 9). For carotenoids, with similar results for growth habits, grass plants had a more positive effect size than woody and herbaceous plants but were non-significant with confidence intervals overlapping with zero (Figure 10a). Among taxonomic groups (monocot and dicot), monocots had a higher effect size than those dicots (Figure 10b). For soluble sugars, grass plants showed the highest effect size followed by woody plants but herbaceous plants were found to be non-significant. This effect was reflected in the taxonomic group, in which monocots showed a higher effect size than dicot plants in AMF inoculated plants under drought stress conditions (Figure 10).

### 3.6. Proline Content

AMF inoculation decreased (1.7%) proline accumulation under drought stress, but its effect was not statistically significant (n = 12; E++ = −0.05; 95% CI, −0.19 to 0.09) where 95% of CIs overlapped with zero (Figure 2). When considering drought intensity, we found that the effect size of proline accumulation increased by 45% for mild stress intensity compared to non-inoculated controls. The effect sizes for moderate and severe stress decreased by 9% and 15%, respectively (*p* =0.03) (Figure 3). Among plant species (Q_B_ = 253; Q_B_/Q_T_ = 0.6; *p* = 0.001), *Lactuca sativa* (n = 9; E++ = 1.19), followed by *Glycine max* (n = 12; E++ = 0.28) showed significantly positive proline accumulation. *S. lycopersicum* (n = 12; E++ = −0.21) followed by *Zea mays* (n = 10; E++ = −0.29) showed negative responses in proline accumulation. Monocot plants were found to possess increased proline accumulation by 1% whereas dicot plants decreased by 6%. Categorical analysis of AMF species (Q_B_ = 33; Q_B_/Q_T_ = 0.21; *p* = 0.015) indicated that AMF species increased proline accumulation under drought stress. Among AMF species, *R. irregularis* showed a more positive response than *F. mosseae* for proline accumulation and was found to be non-significant with confidence intervals overlapping with zero (Figure 4). Among AMF richness (*p* = 0.016, Q_B_ = 6.68; Q_B_/Q_T_ = 0.055), single-species inoculation showed increased (5%) accumulation of proline (df = 95; E++ = 0.06) but was non-significant whereas mixed-species inoculation (df = 30; E++ = −0.40) showed more reduction (33%) in proline accumulation (Figure 5).

## 4. Discussion

Plants have a defense system to defend themselves from drought stress [28,29,30]. The beneficial effects of AMF on plant growth and development are extensively reported in literature reviews, meta-analyses, and research papers [31,32,33,34,35,36,37]. Recently, several studies have been published about the effects of AM fungi on drought stress alleviation, but we still lack consistent estimation of the oxidative stress indicators/ameliorators. Our meta-analysis supports some of the previous results and dispositions. Drought stress induces ROS production and destroys the balance between ROS generation and quenching, resulting in the accumulation of more MDA in plants. MDA, which is a product of peroxidation of lipid membranes in cells, can be used as an indicator of oxidative stress in plants. The results of this study showed that inoculation with AMF significantly reduced the MDA content in plants under moderate and severe drought stress. This confirmed that there is less injury during droughts in AMF-inoculated plants. Thus, the reduced level of MDA in host plants indicates that AMF can reduce damage to the cell membrane. Previous studies have also shown limited accumulation of MDA in mycorrhizal plants under drought stress [9,30,38]. In the present study, it was also found that the accumulation of H_2_O_2_ was reduced by AMF inoculation under drought stress in host plants. Thus, the damage to the cell membrane caused by ROS particularly H_2_O_2_ was lower than that in the NM plants. This indicated that less ROS was accumulated in leaves of inoculated poplars, further confirmed by limited MDA accumulation. This finding is consistent with the results of previous studies [9,30,34].

Furthermore, the percentage of membrane EL, an indicator of cell membrane stability, has been identified as a good indicator of tolerance to drought stress. The low EL values indicate more root hydraulic conductance in mycorrhizal plants. Thus, mycorrhization might increase the plant’s water uptake ability by increasing the effectiveness of the root hydraulic conductivity and transpiration. Similarly, it was reported that AM symbiosis regulated different physiological mechanisms under drought stress. In the present study, carotenoid contents increased significantly more than those of non-inoculated plants under drought stress. Carotenoids are biosynthesized and stored in plastids, where they play essential roles in oxygenic photosynthesis (light-harvesting), photoprotection (detoxification of free radicals generated during photosynthesis), and signaling pathways [39,40]. Moreover, in plants, carotenoids serve as a precursor to the biosynthesis of phytohormones such as abscisic acid and strigolactones. Thus, the carotenoids play a crucial role in regulating several plant developmental and adaptation processes [41]. The higher levels of carotenoid content in AMF-inoculated plants observed in this study represent explicit evidence for the mycorrhizal role in alleviating the adverse effects of drought stress on plants.

Because of the increased level of carotenoids, the precursor for the biosynthesis of ABA accounts for drought stress alleviation. In the present study, mild and moderate levels of drought stress showed an increased level of ABA accumulation than severe levels of drought. It is well known that the endogenous levels of ABA in vegetative plant tissues rise in response to stresses that cause a plant water deficit [42,43,44]. Moreover, a clear relationship between plant ABA content and plant tolerance to drought stress has been described [45,46]. It proves that AMF inoculation was found to be more efficient for the biosynthesis of ABA under severe drought stress. Subsequently, stomatal closure accounts for the minimization of transpirational water loss and root hydraulic conductivity. The sequence of events accounted for above mitigates stress damage through the activation of many stress-responsive genes, which collectively increase plant stress tolerance [42,44,47]. Similar studies have found an increase in ABA content under drought conditions before any physiological parameter changes. Moreover, the combined effects of ABA and AM symbiosis on hydraulic conductivity and aquaporin gene expression regulation was a very essential tool to evaluate the water use efficiency of AMF-inoculated plants under drought stress [11,48].

Under drought stress conditions, plants accumulate some small molecules including organic solutes like soluble sugars and proline [2,22]. In the present study, the soluble sugars of the plants of AMF-inoculated plants increased under drought conditions. The soluble sugar content of AMF-treated seedlings was higher compared to that of NM plants, supporting previous findings [10,49,50]. This might be owed to the fact that AM fungi-improved plant photosynthesis accumulated sugars [51,52]. AM fungi have been reported for induced/enhanced specific plant protein levels under drought stress [53]. In addition, contents of sugars in drought-stressed plants were higher in the roots of AM-inoculated plants when compared with non-AM seedlings. Thus, AM roots represented greater sinks for carbohydrates than non-AM roots. AM fungi take up glucose from the host plants to use for trehalose synthesis, which is needed for sustaining fungal growth and development. The key effect of AM on sugar accumulation has been reported under drought conditions in various studies [10,53,54]. The present meta-analysis also affirms the AM effects in drought stress management. AMF colonization contributes to the accumulation of carbohydrates and the reduction of the osmotic potential of the host plant under water stress. In addition, AM inoculation was shown as an alternative to escape drought stress conditions.

The accumulation of osmotic adjustment substances/markers of oxidative stress, such as proline, is one of the basic adaptations of plants to drought stress. The results showed that mild stress accumulated more proline than moderate and severe stress in the AM plants and this accumulation was decreased with increasing the drought stress levels. This suggests that AMF colonization enhanced the host plant’s drought stress tolerance and thus; plants were less stressed than the NM plants. The results showed that NM plants accumulated more proline than the AM inoculated plants and this accumulation was increased with increasing the drought stress levels. Previous studies also showed that proline accumulation in AM fungi-inoculated plants was lower compared to that in NM plants [11,22,55,56]. Therefore, AMF-treated plants require less proline compared to NM plants. This was consistent with previous studies revealing AMF inoculation and proline content under drought stress conditions [11,55,56,57]. AMF inoculated plants had lower proline levels than non-AM plants when exposed to drought stress conditions, which may be attributed to either greater drought resistance of AM plants or less injury in AM plants grown under drought stress conditions. However, mycorrhizal plants containing higher proline content than non-mycorrhizal plants were also observed in some other studies [53,58]. Moreover, a high proline level may help plants to survive short-time drought and recover from stress. This suggests that AMF colonization enhanced the host plant’s drought stress tolerance and thus; plants were less stressed than the NM plants [50,51,59]. These results suggested that mycorrhizal fungi could alleviate the adverse effects of drought on plants, efficiently. However, proline accumulation in drought-stressed plants varies and depends on plants and drought intensity, showing further research for specificity.

## 5. Conclusions

Several recent studies have demonstrated that AMF inoculation can enhance drought stress tolerance by modulating ROS detoxification and by regulating multiple stress-responsive pathways. Understanding the H_2_O_2_ mechanisms and molecules related to oxidative stress and drought stress tolerance will be valuable for identifying physiological strategies to improve drought stress tolerance in crop plants. The present investigation showed that AMF inoculation significantly alleviated the harmful effects of drought stress on plants grown under different drought stress through different oxidative stress molecules/indicators. The present study clearly showed that AMF inoculation decreased the level of H_2_O_2_, which, in turn, reduced lipid peroxidation by MDA content and membrane stability by the reduced level of EL. Moreover, AMF plants induced high levels of carotenoids, which, in turn, enhanced their soluble sugars and ABA levels better and faster than non-AM plants. Thus, the delicate balance between photosynthesis, transpiration, and root water movement during drought and recovery is ascertained. Finally, AMF-inoculated plants accumulated a reduced level of proline, suggesting that AMF colonization enhanced the host plant’s drought stress tolerance in mycorrhizal plants more than the non-mycorrhizal plants. As a whole, the present study concludes that AMF colonization is responsible for a sequence of events mediated by AMF colonization and accounts for the oxidative stress indicator’s role in mitigating the deleterious effects of drought stress on plant growth.

## Figures and Tables

**Table 1:** Effect of arbuscular mycorrhizal inoculation responses on non-enzymatic antioxidants under drought stress.

Response Variable	Number of Studies	Effect Size	95% Confidence Interval	P _Chi square_
H_2_O_2_	75	−0.2300	−0.3115 to −0.1486	0.99
MDA	124	−0.2316	−0.3669 to −0.0963	1.00
EL	33	−0.3222	−0.4195 to −0.2250	0.01
CAR	39	0.2183	0.1417 to 0.2949	0.0001
ABA	40	−0.1766	−0.4520 to 0.0989	0.28
SS	79	0.1937	0.1268 to 0.2607	0.47
Proline	127	−0.0490	−0.1964 to 0.0984	0.53

## Data Availability

Submitted as a supplementary information.

## Supplementary Materials

The following are available online at https://www.mdpi.com/article/10.3390/plants11192448/s1, Table S1: Dataset, Table S2: References of Meta-analysis.
