# Supplementary material for: Arbuscular Mycorrhizal Fungi Mediated Alleviation of Drought Stress via Non-Enzymatic Antioxidants: A Meta-Analysis
Source: Plants (Basel). 2022 Sep 20;11(19):2448. doi: 10.3390/plants11192448 (PMC9571390; doi:10.3390/plants11192448)
Supplement: Supplementary file 1 [file plants-11-02448-s001.zip › plants-1898527-supplementary (1).pdf]

Appendix 1: References of Meta-analysis

1. Al-Arjani AF, Hashem A, Abd Allah EF. Arbuscular mycorrhizal fungi modulate dynamics tolerance expression to mitigate drought stress in Ephedra foliata Boiss. Saudi J Biol Sci. 2020 Jan;27(1):380-394.
2. Aalipour, H., Nikbakht, A., Etemadi, N., Rejali, F., Soleimani, M., 2020. Biochemical response and interactions between arbuscular mycorrhizal fungi and plant growth promoting rhizobacteria during establishment and stimulating growth of Arizona cypress (Cupressus arizonica G.) under drought stress. Sci. Hortic. 261, 108923.
3. Abbaspour, H., Saeidi-Sar, S., Afshari, H., & Abdel-Wahhab, M. A. (2012). Tolerance of Mycorrhiza infected Pistachio (Pistacia vera L.) seedling to drought stress under glasshouse conditions. Journal of Plant Physiology, 169(7), 704–709.
4. Abdelmalik, A.M.; Alsharani, T.S.; Al-Qarawi, A.A.; Ahmed, A.I.; Aref, I.M. Response of growth and drough ttolerance of Acacia seyal Del. seedlings to arbuscular mycorrhizal fungi.Plant Soil Environ.2020,66, 264–271.
5. Abdelmoneim T.S.; Tarek A.A. Moussa; Almaghrabi O.A.; Hassan S. Alzahrani and Ismail Abdelbagi. Increasing Plant Tolerance to Drought Stress by Inoculation with Arbuscular Mycorrhizal Fungi Life Sci J 2014; 11(1): 10-17.
6. Abdel-Salam, E.; Alatar, A.; El-Sheikh, M.A. Inoculation with arbuscular mycorrhizal fungi alleviates harmful effects of drought stress on damask rose. Saudi J. Biol. Sci. 2018, 25, 1772–1780.
7. Abdi, N.; van Biljon, A.; Steyn, C.; Labuschagne, M.T. Bread Wheat (Triticum aestivum) Responses to Arbuscular Mycorrhizae Inoculation under Drought Stress Conditions. Plants 2021, 10, 1756.
8. Amiri, R., Nikbakht, A., Rahimmalek, M. et al. Variation in the Essential Oil Composition, Antioxidant Capacity, and Physiological Characteristics of Pelargonium graveolens L. Inoculated with Two Species of Mycorrhizal Fungi Under Water Deficit Conditions. J Plant Growth Regul 36, 502–515 (2017).
9. Anli M, Baslam M, Tahiri A, Raklami A, Symanczik S, Boutasknit A, Ait-El-Mokhtar M, Ben-Laouane R, Toubali S, Ait Rahou Y, Ait Chitt M, Oufdou K, Mitsui T, Hafidi M and Meddich A (2020) Biofertilizers as Strategies to Improve Photosynthetic Apparatus, Growth, and Drought Stress Tolerance in the Date Palm. Front. Plant Sci. 11:516818.
10. Armada E, Azcón R, López-Castillo OM, Calvo-Polanco M, Ruiz-Lozano JM. (2015). Autochthonous arbuscular mycorrhizal fungi and Bacillus thuringiensis from a degraded Mediterranean area can be used to improve physiological traits and performance of a plant of agronomic interest under drought conditions. Plant Physiol Biochem. 90:64-74.
11. Aroca R, Aguacil MM, Vernieri P, Ruiz-Lozano JM (2008) Plant responses to drought stress and exogenous ABA application are differently modulated by mycorrhization in tomato and an ABA-deficient mutant (sitiens). Microb Ecol 56:704–719
12. Asensio, D., Rapparini, F., Pe˜nuelas, J., 2012. AM fungi root colonization increases the production of essential isoprenoids vs. nonessential isoprenoids especially under

drought stress conditions or after jasmonic acid application. Phytochemistry 77, 149–161.

13. Asrar, A. W., & Elhindi, K. M. (2011). Alleviation of drought stress of marigold (Tagetes erecta) plants by using arbuscular mycorrhizal fungi. Saudi journal of biological sciences, 18(1), 93–98.
14. Asrar, A.A., Abdel-Fattah, G.M. & Elhindi, K.M. Improving growth, flower yield, and water relations of snapdragon (Antirhinum majus L.) plants grown under well-watered and water-stress conditions using arbuscular mycorrhizal fungi. Photosynthetica 50, 305–316 (2012).
15. Azmat R and Moin, S 2019. The remediation of drought stress under VAM inoculation through proline chemical transformation action. Journal of Photochemistry and Photobiology B: Biology, 193, 155-161.
16. Bagheri, V., Shamshiri, M.H., Malaei, H., & Salehi, H. (2019). The role of inoculum identity for growth, photosynthesis, and chlorophyll fluorescence of zinnia plants by arbuscular mycorrhizal fungi under varying water regimes. Photosynthetica, 57(2), 409-419.
17. Bakr, J., Pék, Z., Helyes, L., Posta, K. (2018). Mycorrhizal Inoculation Alleviates Water Deficit Impact on Field-Grown Processing Tomato. Polish Journal of Environmental Studies, 27(5), 1949-1958.
18. Barnawal, D., Maji, D., Bharti, N. et al. ACC Deaminase-Containing Bacillus subtilis Reduces Stress Ethylene-Induced Damage and Improves Mycorrhizal Colonization and Rhizobial Nodulation in Trigonella foenum-graecum Under Drought Stress. J Plant Growth Regul 32, 809–822 (2013).
19. Begum N, Ahanger MA, Su Y, Lei Y, Mustafa NSA, Ahmad P, Zhang L. Improved Drought Tolerance by AMF Inoculation in Maize (Zea mays) Involves Physiological and Biochemical Implications. Plants (Basel). 2019, 8(12):579.
20. Begum, N., Ahanger, M.A., Zhang, L., 2020. AMF inoculation and phosphorus supplementation alleviates drought induced growth and photosynthetic decline in Nicotiana tabacum by up-regulating antioxidant metabolism and osmolyte accumulation. Environ. Exp. Bot. 176, 104088
21. J. BELTRANO and M.G. RONCO (2008). Improved tolerance of wheat plants (Triticum aestivum L.) to drought stress and rewatering by the arbuscular mycorrhizal fungus Glomus claroideum: Effect on growth and cell membrane stability. Braz. J. Plant Physiol., 20(1):29-37.
22. Benhiba, L., Fouad, M. O., Essahibi, A., Ghoulam, C., and Qaddoury, A. (2015). Arbuscular mycorrhizal symbiosis enhanced growth and antioxidant metabolism in date palm subjected to long-term drought. Trees 29, 1725–1733.
23. M. J. Bompadre, M. C. Rios de Molina, R. P. Colombo et al., “Differential efficiency of two strains of the arbuscular mycorrhizal fungus Rhizophagus irregularis on olive (Olea europaea) plants under two water regimes,” Symbiosis, vol. 61, no. 2, pp. 105–112, 2013.
24. Boutasknit, A.; Baslam, M.; Ait-El-Mokhtar, M.; Anli, M.; Ben-Laouane, R.; Douira, A.; El Modafar, C.; Mitsui, T.; Wahbi, S.; Meddich, A. Arbuscular Mycorrhizal Fungi Mediate Drought Tolerance and Recovery in Two Contrasting Carob (Ceratonia siliqua

L.) Ecotypes by Regulating Stomatal, Water Relations, and (In)Organic Adjustments. Plants 2020, 9, 80.
25. Calvo-Polanco M, Sánchez-Castro I, Cantos M, García JL, Azcón R, Ruiz-Lozano JM, Beuzón CR, Aroca R. Effects of different arbuscular mycorrhizal fungal backgrounds and soils on olive plants growth and water relation properties under well-watered and drought conditions. Plant Cell Environ. 2016 Nov;39(11):2498-2514.
26. Chen W, Meng P, Feng H, Wang C. Effects of Arbuscular Mycorrhizal Fungi on Growth and Physiological Performance of Catalpa bungei C.A.Mey. under Drought Stress. Forests. 2020; 11(10):1117
27. Chitarra, W.; Pagliarani, C.; Maserti, B.; Lumini, E.; Siciliano, I.; Cascone, P.; Schubert, A.; Gambino, G.; Balestrini, R.; Guerrieri, E. Insights on the impact of arbuscular mycorrhizal symbiosis on tomato tolerance to water stress. Plant Physiol. 2016, 171, 1009–1023.
28. Doubková, P., Vlasáková, E. & Sudová, R. Arbuscular mycorrhizal symbiosis alleviates drought stress imposed on Knautia arvensis plants in serpentine soil. Plant Soil 370, 149–161 (2013).
29. Fouad, O. M., Essahibi, A., Benhiba, L., and Qaddoury, A. (2014). Effectiveness of arbuscular mycorrhizal fungi in the protection of olive plants against oxidative stress induced by drought. Span. J. Agric. Res. 12, 763–771.
30. Gong, M., You, X. & Zhang, Q. Effects of Glomus intraradices on the growth and reactive oxygen metabolism of foxtail millet under drought. Ann Microbiol 65, 595–602 (2015).
31. Grümberg, B. C., María, U. C., Shroeder, A., Vargas-Gil, S., Luna, C. M. (2015). The role of inoculum identity in drought stress mitigation by arbuscular mycorrhizal fungi in soybean. Biol. Fert. Soils 51, 1–10.
32. He F, Sheng M and Tang M (2017) Effects of Rhizophagus irregularis on Photosynthesis and Antioxidative Enzymatic System in Robinia pseudoacacia L. under Drought Stress. Front. Plant Sci. 8:183.
33. He, J. D., Zou, Y. N., Wu, Q. S., and Kuča, K. (2020). Mycorrhizas enhance drought tolerance of trifoliate orange by enhancing activities and gene expression of antioxidant enzymes. Sci. Hortic. 262, 108745
34. Huang, Z., Zou, Z., He, C. et al. Physiological and photosynthetic responses of melon (Cucumis melo L.) seedlings to three Glomus species under water deficit. Plant Soil 339, 391–399 (2011).
35. Huang, Y. M., Zou, Y. N., and Wu, Q. S. (2017). Alleviation of drought stress by mycorrhizas is related to increased root H2O2 efflux in trifoliate orange. Sci. Rep. 7, 423–435.
36. Huang D, Ma M, Wang Q, Zhang M, Jing G, Li C, Ma F. Arbuscular mycorrhizal fungi enhanced drought resistance in apple by regulating genes in the MAPK pathway. Plant Physiol Biochem. 2020, 149:245-255.
37. Huang, D., Wang, Q., Zhang, Z. et al. Silencing MdGH3-2/12 in apple reduces drought resistance by regulating AM colonization. Hortic Res 8, 84 (2021).

38. Igiehon NO, Babalola OO. Rhizosphere Microbiome Modulators: Contributions of Nitrogen Fixing Bacteria towards Sustainable Agriculture. Int J Environ Res Public Health. 2018 Mar 23;15(4):574.
39. Jadrane, I. et al. Inoculation with selected indigenous mycorrhizal complex improves Ceratonia siliqua’s growth and response to drought stress. Saudi J. Biol. Sci. 28, 825–832 (2021).
40. Kong Jing, Pei Zongping, Du Min, Sun Gan, Zhang Xin. Effects of arbuscular mycorrhizal fungi on the drought resistance of the mining area repair plant Sainfoin. Int. J. Min. Sci. Tech. 2014;24(4):485–489.
41. Krishna, M. S. R. (2018). Arbuscular mycorrhizal symbiosis alters morphological and biochemical indices in hot pepper (Capsicum annuum L.) under drought stress. International Journal of Green Pharmacy (IJGP), 12(02).
42. Kamali S, Mehraban A (2020) Effects of Nitroxin and arbuscular mycorrhizal fungi on the agro-physiological traits and grain yield of sorghum (Sorghum bicolor L.) under drought stress conditions. PLoS ONE 15(12): e0243824.
43. Lahbouki S., Ben-Laouane R., Anli M., Boutasknit A., Ait-Rahou Y., Ait-El-Mokhtar M., et al. (2022). Arbuscular mycorrhizal fungi and/or organic amendment enhance the tolerance of prickly pear (Opuntia ficus-indica) under drought stress. J. Arid Environ. 199:104703.
44. Lee BR, Muneer S, Jung WJ, Avice JC, Ourry A, Kim TH. Mycorrhizal colonization alleviates drought-induced oxidative damage and lignification in the leaves of drought-stressed perennial ryegrass (Lolium perenne). Physiol Plant. 2012 Jul;145(3):440-9.
45. Liu Z, Li Y, Ma L, Wei H, Zhang J, He X, Tian C. Coordinated regulation of arbuscular mycorrhizal fungi and soybean MAPK pathway genes improved mycorrhizal soybean drought tolerance. Mol Plant Microbe Interact. 2015; 28(4):408-19.
46. Liu, J., Guo, C., Chen, Z. L., He, J. D., & Zou, Y. N. (2016). Mycorrhizal inoculation modulates root morphology and root phytohormone responses in trifoliate orange under drought stress. Emirates Journal of Food and Agriculture, 251-256.
47. Liu, L., Li, D., Ma, Y., Shen, H., Zhao, S., & Wang, Y. (2021). Combined application of arbuscular mycorrhizal fungi and exogenous melatonin alleviates drought stress and improves plant growth in tobacco seedlings. Journal of Plant Growth Regulation, 40(3), 1074-1087.
48. Liao X, Chen J, Guan R, Liu J, Sun Q. Two Arbuscular Mycorrhizal Fungi Alleviates Drought Stress and Improves Plant Growth in Cinnamomum migao Seedlings. Mycobiology. 2021; 49(4):396-405.
49. Liang, S. M., Jiang, D. J., Miao-Miao, X. I. E., Ying-Ning, Z. O. U., Qiang-Sheng, W. U., & Kamil, K. U. Č. A. (2021). Physiological responses of mycorrhizal symbiosis to drought stress in white clover. Notulae Botanicae Horti Agrobotanici Cluj-Napoca, 49(1), 12209-12209.
50. Mannino, G.; Nerva, L.; Gritli, T.; Novero, M.; Fiorilli, V.; Bacem, M.; Bertea, C.M.; Lumini, E.; Chitarra, W.; Balestrini, R. Effects of Different Microbial Inocula on Tomato Tolerance to Water Deficit. Agronomy 2020, 10, 170.
51. Mena-Violante, H. G., Ocampo-Jimenez, O., Dendooven, L., Martinez-Soto, G., Gonzalez-Castafeda, J., Davies, F. T., et al. (2006). Arbuscular mycorrhizal fungi

enhance fruit growth and quality of chile ancho Capsicum annuum L. cv San Luis plants exposed to drought. Mycorrhiza 16, 261–267.

52. Mo, Y. L. et al. Regulation of plant growth, photosynthesis, antioxidation and osmosis by an arbuscular mycorrhizal fungus in watermelon seedlings under well-watered and drought conditions. Front. Plant Sci. 7, 644 (2016).
53. Moradtalab, N., Hajiboland, R., Aliasgharzad, N., Hartmann, T. E., & Neumann, G. (2019). Silicon and the association with an arbuscular-mycorrhizal fungus (*Rhizophagus clarus*) mitigate the adverse effects of drought stress on strawberry. Agronomy, 9(1), 41.
54. Kobra Zeighami Nejad, Mostafa Ghasemi, Mansoore Shamili & Gholam Reza Damizadeh (2020) Effect of Mycorrhiza and Vermicompost on Drought Tolerance of Lime Seedlings (*Citrus aurantifolia* Cv. Mexican Lime), International Journal of Fruit Science, 20:3, 646-657.
55. Ouledali, S., Ennajeh, M., Zrig, A., Gianinazzi, S., & Khemira, H. (2019). Estimating the contribution of arbuscular mycorrhizal fungi to drought tolerance of potted olive trees (*Olea europaea*). Acta Physiologiae Plantarum, 40(5), 1-13.
56. Porcel R, Ruiz-Lozano JM (2004) Arbuscular mycorrhizal influence on leaf water potential, solute accumulation and oxidative stress in soybean plants subjected to drought stress. J Exp Bot 55:1743–1750.
57. Quiroga G, Erice G, Aroca R, Chaumont F and Ruiz-Lozano JM (2017) Enhanced Drought Stress Tolerance by the Arbuscular Mycorrhizal Symbiosis in a Drought-Sensitive Maize Cultivar Is Related to a Broader and Differential Regulation of Host Plant Aquaporins than in a Drought-Tolerant Cultivar. Front. Plant Sci. 8:1056
58. Ren, A. T., Zhu, Y., Chen, Y. L., Ren, H. X., Li, J. Y., Abbott, L. K., & Xiong, Y. C. (2019). Arbuscular mycorrhizal fungus alters root-sourced signal (abscisic acid) for better drought acclimation in Zea mays L. seedlings. Environmental and Experimental Botany, 167, 103824.
59. Ruiz-Lozano JM, Azcón R, Gómez M (1995) Effects of arbuscular mycorrhizal Glomus species on drought tolerance: physiological and nutritional plant responses. Appl Environ Microbiol 61:456–460.
60. Ruíz-Lozano, J.M., del Carmen Perálvarez, M., Aroca, R. et al. The application of a treated sugar beet waste residue to soil modifies the responses of mycorrhizal and non mycorrhizal lettuce plants to drought stress. Plant Soil 346, 153 (2011).
61. Ruiz-Lozano, J. M. et al. Arbuscular mycorrhizal symbiosis induces strigolactone biosynthesis under drought and improves drought tolerance in lettuce and tomato. Plant Cell Environ. 39, 441–452 (2016).
62. Ruíz-Sánchez M, Armada E, Muñoz Y, García de Salamone IE, Aroca R, Ruíz-Lozano JM, Azcón R. Azospirillum and arbuscular mycorrhizal colonization enhance rice growth and physiological traits under well-watered and drought conditions. J Plant Physiol. 2011 Jul 1;168(10):1031-7.
63. Sepahvand, T., Etemad, V., Matinizade, M., & Shirvany, A. (2021). Symbiosis of AMF with growth modulation and antioxidant capacity of Caucasian Hackberry (Celtis Caucasica L.) seedlings under drought stress. Central Asian Journal of Environmental Science and Technology Innovation, 2(1), 20-35.

64. Sohrabi, Y., Heidari, G., Weisany, W. et al. Some physiological responses of chickpea cultivars to arbuscular mycorrhiza under drought stress. Russ J Plant Physiol 59, 708–716 (2012).
65. Tian, Y. H., Lei, Y. B., Zheng, Y. L., & Cai, Z. Q. (2013). Synergistic effect of colonization with arbuscular mycorrhizal fungi improves growth and drought tolerance of Plukenetia volubilis seedlings. Acta Physiologiae Plantarum, 35(3), 687-696.
66. Tuo X-Q et al. Alleviation of Drought Stress in White Clover after Inoculation with Arbuscular Mycorrhizal Fungi. Not Bot Horti Agrobo, 2017, 45(1):220-224
67. Tyagi, J., Varma, A., & Pudake, R. N. (2017). Evaluation of comparative effects of arbuscular mycorrhiza (Rhizophagus intraradices) and endophyte (Piriformospora indica) association with finger millet (Eleusine coracana) under drought stress. European Journal of Soil Biology, 81, 1-10.
68. Wu QS, Xia RX (2006) Arbuscular mycorrhizal fungi influence growth, osmotic adjustment and photosynthesis of citrus under well-watered and water stress conditions. J Plant Physiol163:417–425.
69. Wu QS, Zou YN, Xia RX. Effect of Glomus versiforme inoculation on reactive oxygen metabolism of Citrus tangerine leaves exposed to water stress Front. Agric. China 2007, 1(4): 438–443.
70. Wu, Q. S., Zou, Y. N., Xia, R. X., & Wang, M. Y. (2007). Five Glomus species affect water relations of Citrus tangerine during drought stress. Bot Stud, 48(2), 147-154.
71. Wu Q.S., Zou Y.N. (2009). Mycorrhiza has a direct effect on reactive oxygen metabolism of drought-stressed citrus. Plant Soil Environ., 55: 436-442.
72. Wu, H. H., Zou, Y. N., Rahman, M. M., Ni, Q. D., & Wu, Q. S. (2017). Mycorrhizas alter sucrose and proline metabolism in trifoliate orange exposed to drought stress. Scientific Reports, 7(1), 1-10.
73. Wu, Q.S.; He, J.D.; Srivastava, A.K.; Zou, Y.N.; Kuˇca, K. Mycorrhizas enhance drought tolerance of citrus by altering root fatty acid compositions and their saturation levels. Tree Physiol. 2019, 39, 1149–1158
74. Xu, L., Li, T., Wu, Z., Feng, H., Yu, M., Zhang, X., & Chen, B. (2018). Arbuscular mycorrhiza enhances drought tolerance of tomato plants by regulating the 14-3-3 genes in the ABA signaling pathway. Applied soil ecology, 125, 213-221.
75. Yaghoubian, Y., Goltapeh, E. M., Pirdashti, H., Esfandiari, E., Feiziasl, V., Dolatabadi, H. K., ... & Hassim, M. H. (2014). Effect of Glomus mosseae and Piriformospora indica on growth and antioxidant defense responses of wheat plants under drought stress. Agricultural Research, 3(3), 239-245.
76. Yang, Q.; Zhao, Z.; Bai, Z.; Hou, H.; Yuan, Y.; Guo, A.; Li, Y. Effects of mycorrhizae and water conditions on perennial ryegrass growth in rare earth tailings. RSC Adv. 2019, 9, 10881–10888.
77. Zhang, Y., Zhong, C.L., Chen Y., Chen Z., Jiang, Q.B., Wu C., Pinyopusarerk, K. (2010). Improving drought tolerance of *Casuarina equisetifolia* seedlings by arbuscular mycorrhizas under glasshouse conditions. New Forests 40:261–271.
78. Zhang, Z., Zhang, J., & Huang, Y. (2014). Effects of arbuscular mycorrhizal fungi on the drought tolerance of *Cyclobalanopsis glauca* seedlings under greenhouse conditions. New forests, 45(4), 545-556.

79. Zhang, Y., Yao, Q., Li, J., Hu, Y., & Chen, J. (2015). Growth response and nutrient uptake of *Eriobotrya japonica* plants inoculated with three isolates of arbuscular mycorrhizal fungi under water stress condition. Journal of Plant Nutrition, 37(5), 690-703.
80. Zhang, T., Hu, Y., Zhang, K., Tian, C., & Guo, J. (2018a). Arbuscular mycorrhizal fungi improve plant growth of *Ricinus communis* by altering photosynthetic properties and increasing pigments under drought and salt stress. Industrial Crops and Products, 117, 13-19.
81. Zhang, F., Jia-Dong, H. E., Qiu-Dan, N. I., Qiang-Sheng, W. U., & Ying-Ning, Z. O. U. (2018b). Enhancement of drought tolerance in trifoliate orange by mycorrhiza: changes in root sucrose and proline metabolisms. Notulae Botanicae Horti Agrobotanici Cluj-Napoca, 46(1), 270-276.
82. Zhang, Z., Zhang, J., Xu, G., Zhou, L., & Li, Y. (2019). Arbuscular mycorrhizal fungi improve the growth and drought tolerance of *Zenia insignis* seedlings under drought stress. New Forests, 50(4), 593-604.
83. Zhu, X., Song, F., & Liu, S. (2011). Arbuscular mycorrhiza impacts on drought stress of maize plants by lipid peroxidation, proline content and activity of antioxidant system. J Food Agric Environ, 9(2), 583-587
84. Zou Y-N, Wu Q-S, Huang Y-M, Ni Q-D, He X-H (2013) Mycorrhizal-Mediated Lower Proline Accumulation in Poncirus trifoliata under Water Deficit Derives from the Integration of Inhibition of Proline Synthesis with Increase of Proline Degradation. PLoS ONE 8(11): e80568.
